# Laparoscopic and Robot-Assisted Laparoscopic Management of Iatrogenic Ureteral Strictures: Preliminary Experience

**DOI:** 10.3390/life15040645

**Published:** 2025-04-14

**Authors:** Roxana Andra Coman, Bogdan Petrut

**Affiliations:** 1 Department of Urology, MedLife Humanitas Hospital, 400664 Cluj-Napoca, Romania; 2 Department of Urology, “Iuliu Hatieganu” University of Medicine and Pharmacy, 400012 Cluj-Napoca, Romania

**Keywords:** iatrogenic ureteral stricture, laparoscopic ureteral repair, robotic ureteral repair, ureteral stricture, ureteroureterostomy, ureteroscopy

## Abstract

Iatrogenic ureteral strictures are uncommon but challenging to manage. We present our expertise in laparoscopic and robot-assisted laparoscopic ureteroureterostomy (LUU and RAUU) for lumbar and iliac strictures and laparoscopic ureteral reimplantation for pelvic strictures. A descriptive study was conducted on nine adult patients who underwent minimally invasive procedures. Six had lumbar or iliac ureteral strictures—five due to ureterorenoscopy and one following pancreaticoduodenectomy for pancreatic cancer. Three developed pelvic strictures after ureterorenoscopy. Preoperative evaluation included a medical history review, abdominal ultrasound, and CT scan. Success was characterized by the absence of symptoms and the lack of obstruction on follow-up imaging at one year. All procedures were technically feasible, with a median operating time of 105 min and a median hospital stay of four days. No major complications occurred. One patient experienced ureteral stricture recurrence following a laparoscopic approach for a lumbar stricture, and required a permanent double-J stent. At a median follow-up of 38 months, 88.88% of patients remained asymptomatic with preserved renal function. Our findings suggest that robotic and laparoscopic ureteral reconstruction performed by experienced surgeons at a tertiary center is a safe and effective option with a low complication rate.

## 1. Introduction

Ureteral reconstruction is a complex challenge in reconstructive urology, particularly in cases with prior ureteral surgery. Fibrosis and obliterated surgical planes often complicate the procedure, making meticulous preoperative planning essential. Successful management of ureteral complications requires a thorough patient history, detailed diagnostic evaluation, and a comprehensive understanding of any anatomical alterations from previous interventions. The approach to reconstruction varies depending on the location of the stricture (upper, middle, or lower ureter), with it being crucial to ensure adequate blood supply in order to prevent complications such as ureteral necrosis or recurrent strictures. Consequently, techniques that facilitate wide, tension-free anastomoses with sufficient vascularization are imperative [1].

Ureteral strictures are observed in approximately 3.5% of cases following endoscopic procedures for stone treatment [2]. Depending on the location, there may be several reconstruction options. Middle ureteral strictures present a particular challenge, as their repair may not be feasible using bladder or renal pelvis mobilization alone. Long strictures can also be difficult to bridge, limiting surgical options [1].

The first laparoscopic ureteroureterostomy (LUU) was performed by Nezhat and colleagues in 1992 [3], followed by the first laparoscopic ureteral reimplantation by Ehrlich and Gershman in 1993 [4]. While LUU may be a challenging procedure in terms of suturing, it could serve as a viable option for open surgery. Since its introduction, LUU has become one of the most frequently described laparoscopic ureteral reconstructive procedures. Success rates have reached over 90% [5]. The advent of robotic-assisted surgery has further refined this approach. Robot-assisted laparoscopic ureteroureterostomy (RAUU) represents an alternative to the laparoscopic approach for reduced ureteral resection and intracorporeal suturing, particularly for surgeons with minimal laparoscopic experience [6].

Performing minimally invasive ureteroureterostomy on the lumbar and iliac segments can be demanding because the site and size of the stricture have to be precisely identified during the surgery. Ensuring anastomosis on healthy, well-vascularized tissue without tension is critical to achieving durable outcomes.

This study aims to present our experience in managing iatrogenic ureteral strictures using a minimally invasive approach, evaluating surgical feasibility, outcomes, and complications.

## 2. Materials and Methods

Between 2014 and 2023, nine patients with iatrogenic ureteral stricture were treated. Informed consent was obtained from all participants. Eight patients developed ureteral stricture secondary to ureterorenoscopy with laser lithotripsy for stone treatment, and one after pancreaticoduodenectomy performed for pancreatic cancer. Patients were diagnosed with urinary tract obstruction based on their symptoms and radiographic findings. Initial imaging included an abdominal ultrasound, followed by computed tomography (CT) to confirm the diagnosis. The indications for LUU and RAUU were lumbar and iliac strictures. Out of the six patients, four had lumbar ureteral strictures, and two patients had iliac ureteral stenosis. Three developed pelvic strictures following ureterorenoscopy, and underwent laparoscopic ureteral reimplantation via an extravesical approach.

Assessment of the diseased ureteral segment and determination of the length of the potential defect to be bridged are essential for the planning of the required repair and to aid in preoperative counseling. The primary clinical outcome was to restore a patent ureter and treat symptoms. Technical success was defined as completing the laparoscopic or robotic procedure, while clinical success was defined as the absence of the stricture, recovery, or preservation of renal function, and no symptoms at the last follow-up. For the follow-up, we performed abdominal ultrasound, urinalysis, and urine culture at 1 month, and CT or urography and abdominal ultrasound at every 6 months. We evaluated the complications using the Clavien Dindo classification [7].

Patients were treated with different laparoscopic and robotic techniques according to the level of stenosis: total ureterectomy or partial ureterectomy with end-to-end anastomosis for lumbar and iliac strictures, and ureteral reimplantation for distal ureteral strictures.

### 2.1. LUU and RAUU

A transperitoneal approach was used. The Da Vinci Xi robotic system (Intuitive Surgical, Sunnyvale, CA, USA) was utilized for one patient. After intravenous antibiotic administration and induction of general anesthesia, a 16Fr urethral catheter was inserted into the bladder. The patient was positioned in a right lateral decubitus position at 45°. We established the pneumoperitoneum using a mini-open technique, following the Hasson approach. The surgeon placed the camera trocar along the left lateral edge of the rectus sheath, cephalad to the umbilicus. Two 8 mm robotic trocars were inserted: one at the midpoint between the anterior superior iliac spine and the umbilicus and another on the pararectal line 2–3 cm below the costal arch. An assistant port of 12 mm was placed caudal and inferior to the camera trocar. The surgical procedure began with docking the robotic surgical system. The non-dominant hand was equipped with bipolar Maryland forceps, while the dominant hand was equipped with monopolar Endoscissors. A 30° optic was used.

**Figure 1:**
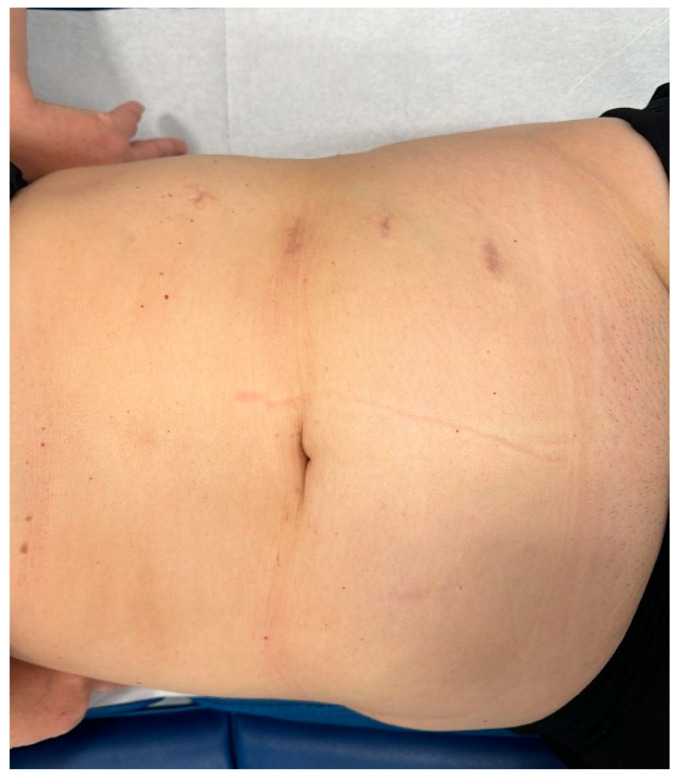
Trocar positioning for left-side laparoscopic transperitoneal ureteroureterostomy.

For laparoscopic cases, the same transperitoneal approach and patient positioning were used. Laparoscopic ports were placed along the midclavicular line, from two fingerbreadths below the costal margin to 2–3 fingerbreadths above the iliac crest (Figure 1). A 30° optic was used in all cases. This configuration allowed us to change instrument positions during surgery.

**Figure 2:**
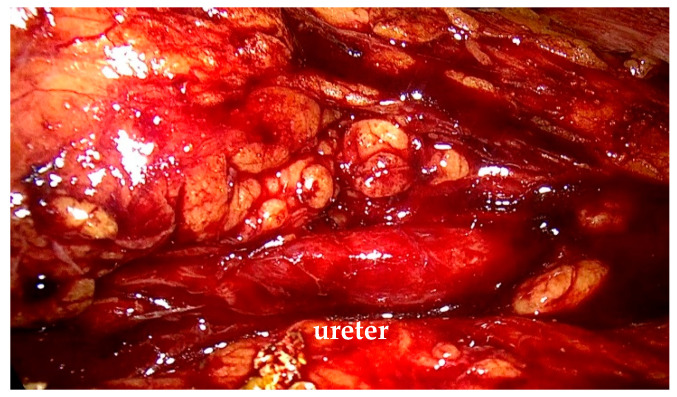
Isolation of the ureter with the stenotic segment and the dilatated upper ureter.

To mobilize the colon, we incised the white line of Toldt, exposed Gerota’s fascia, and identified the ureter (Figure 2).

**Figure 3:**
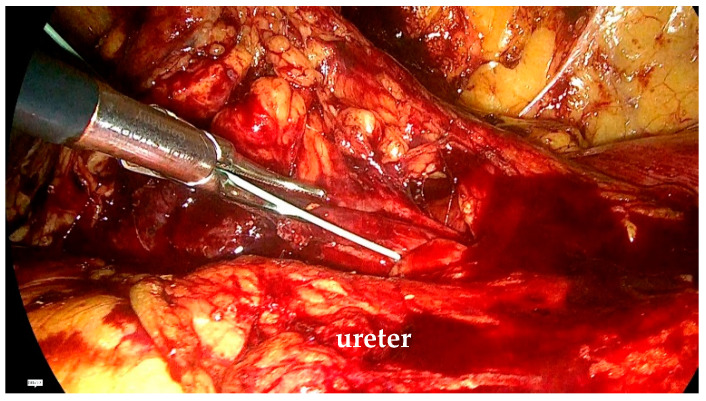
Descending the ureteral stent to the urinary bladder.

**Figure 4:**
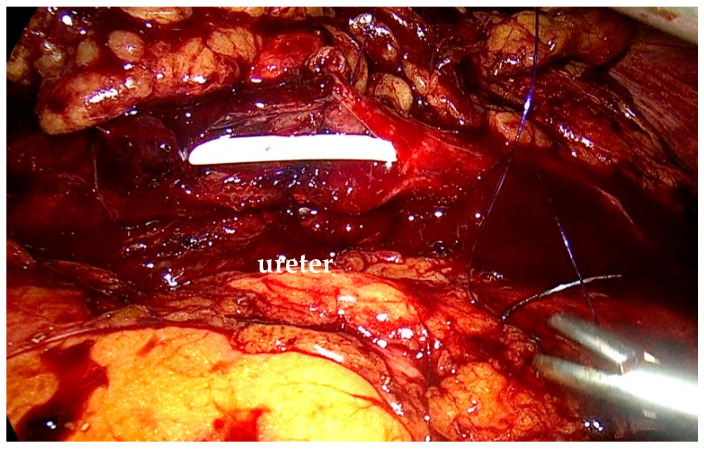
The ureteral stent is correctly placed, and the posterior suture is performed.

The stenotic segment was excised, and the ureter was opened to confirm the presence of healthy tissue. Spatulation of the ureteral edges (≥5 mm) was performed to optimize anastomotic healing and reduce the risk of restenosis. End-to-end ureteroureterostomy was performed using interrupted 4-0 polyglactin sutures. The posterior anastomosis was completed first. Before finishing the anterior anastomosis, a double-J stent was placed under direct visualization through a peripheral venous catheter inserted via the abdominal wall. A 0.889 mm (0.035-inch) polytetrafluoroethylene wire was advanced through the distal ureter into the bladder until resistance was encountered (Figure 3). The proximal end of the stent was positioned in the renal pelvis before finalizing the anastomosis (Figure 4). A drain was left in place at the end of the procedure.

For excised segments under 3 cm, we performed a direct anastomosis as described above. However, for segments longer than 3 cm, we mobilized the bladder and performed a psoas hitch using a 2-0 polyglactin suture, as well as mobilizing the ipsilateral kidney. We sutured through the renal capsule at the superior pole using 2 poliglecaprone sutures, and fixed the ends tightly at the skin using a compress in the lower abdomen, to avoid tension in the anastomosis. The suture that caused traction of the kidney was removed on days 2–3 after surgery. This was used in 2 laparoscopic cases.

To prevent tension in the anastomosis during the robotic procedure, we utilized three 4.0 polyglactin sutures arranged in a triangular pattern. These sutures were gradually used to approach the healthy ureteral segments, with Hem-o-lock clips employed to avoid tension in the anastomosis. The remaining sutures were then applied, and at the end, the Hem-o-lock clips were removed.

In one case, a Heineke–Mikulicz-type repair was performed by cutting along the stenotic site and then closing the wound across the width with interrupted 4-0 polyglactin sutures.

### 2.2. Ureteral Reimplantation for Distal Ureteral Strictures

Laparoscopic ureteral reimplantation was performed using a transperitoneal approach. After induction, the patient was placed in a dorsal lithotomy position. A transperitoneal approach was used. Trocars were placed using the Hasson technique. The first trocar was placed in the midline cranial to the umbilicus and was the optic trocar. The laparoscopic trocars were placed similarly to in a laparoscopic cystectomy approach to facilitate bladder mobilization. The other four trocars were placed symmetrically. Two working trocars of 10 mm were placed on the midclavicular line at the level of the umbilicus, and the other two working trocars of 10 mm and 5 mm, respectively, were placed on the left side of the patient on the right side of the patient above the ipsilateral anterior iliac spine. After trocar placement, the patient was placed in the Trendelenburg position.

**Figure 5:**
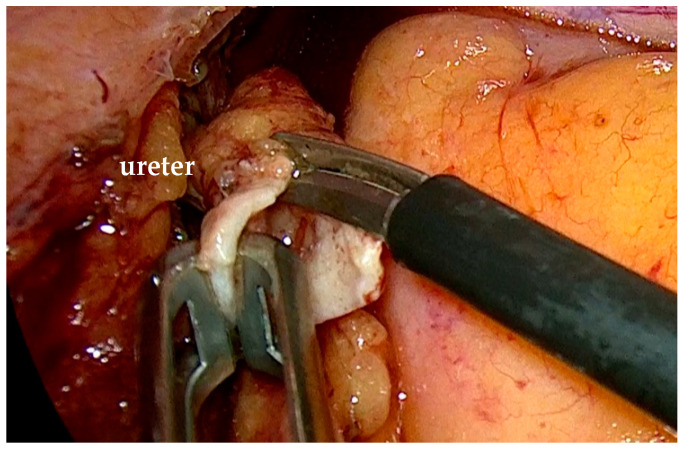
The healthy part of the ureter is inspected and spatulated.

The white line of Toldt was incised, and the colon was medially mobilized to expose the ureter. The ureter was circumferentially dissected, while preserving its blood supply. We mobilized the ureter to the stenotic segment, then inspected and spatulated the remaining healthy ureter. A double-J stent was inserted up to the renal pelvis (Figure 5).

**Figure 6:**
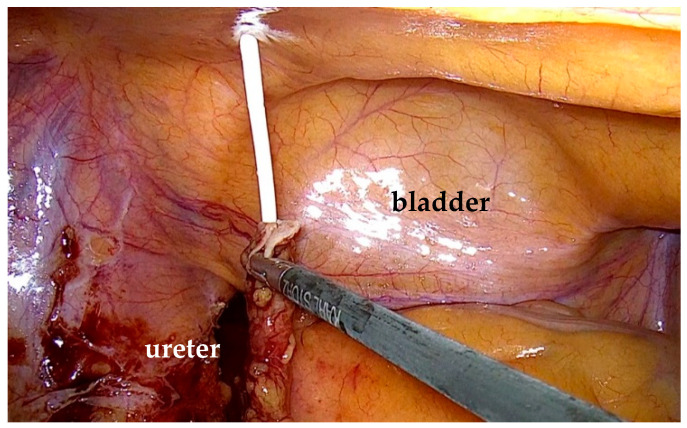
Ureteral stent insertion towards the renal pelvis.

A double-J stent is inserted up to the renal pelvis (Figure 6).

**Figure 7:**
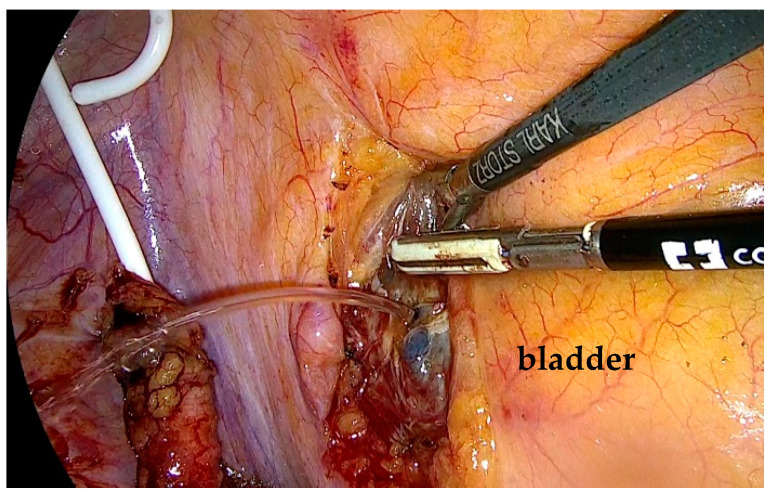
Cistotomy at the ureteral reimplantation site.

The bladder was then filled with 200 to 300 mL of saline and a cystotomy was performed at the dome (Figure 7). Ureterocystostomy anastomosis was completed using resorbable sutures.

**Figure 8:**
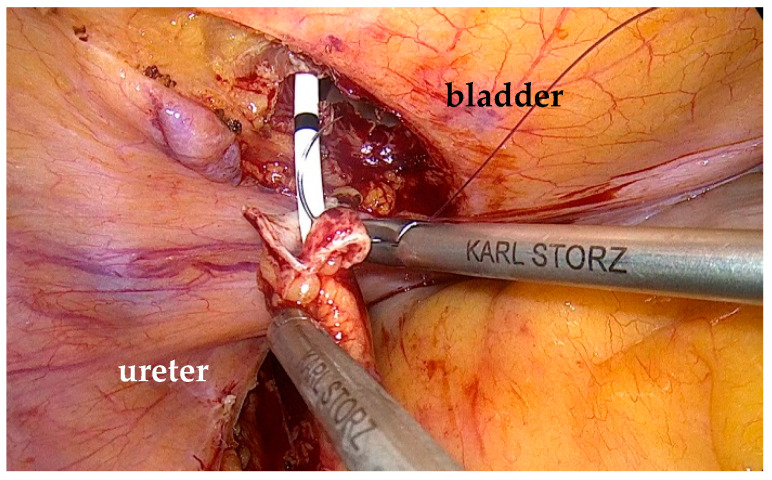
Anasthomosis between the ureter and the bladder—extravesical approach.

**Figure 9:**
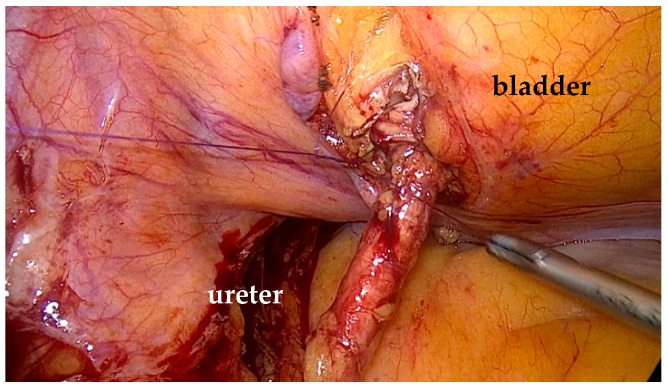
The final aspect of the anastomosis.

The ureter was inserted through a tunnel in the bladder wall, and the detrusor muscle was stitched over it with interrupted 4–0 polyglactin sutures through an extravesical approach (Figure 8 and Figure 9).

We assessed the creatinine level in the drain output, and removed the drainage if there was no increase in output and the creatinine level was low. In LUU and RAUU cases, the bladder catheter was removed 2 weeks after surgery, and the ureteral stents were removed 6 months after surgery. However, in the ureteral reimplantation procedure, we also placed a suprapubic catheter, which was removed 3 weeks after surgery, and the bladder catheter was kept only during the hospitalization period. The ureteral stents were also removed 6 months after surgery.

## 3. Results

A total of nine patients underwent minimally invasive ureteral reconstruction: one via a robotic approach and eight via a laparoscopic approach.

Table 1 summarizes the demographic and preoperative clinical characteristics of the patients. The minimally invasive approach was technically feasible in all cases, with no need for conversion to open surgery. The median ureteral stricture length, measured via CT scan, was 19 mm (range: 5–40 mm). Table 2 presents intraoperative and postoperative data. The median operative time was 105 min, and the median hospital stay was 4 days. Catheterization time and hospitalization duration were comparable among all patients, with no significant perioperative complications.

**Figure 10:**
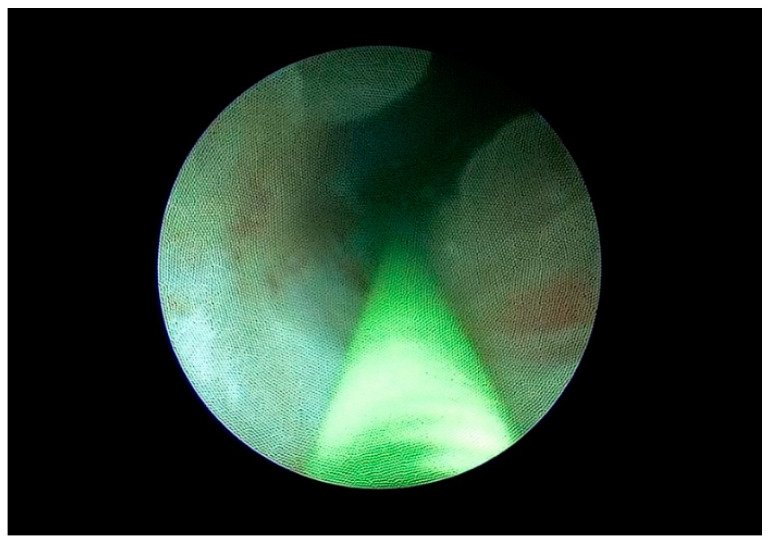
The endoscopic aspect of LUU.

**Figure 11:**
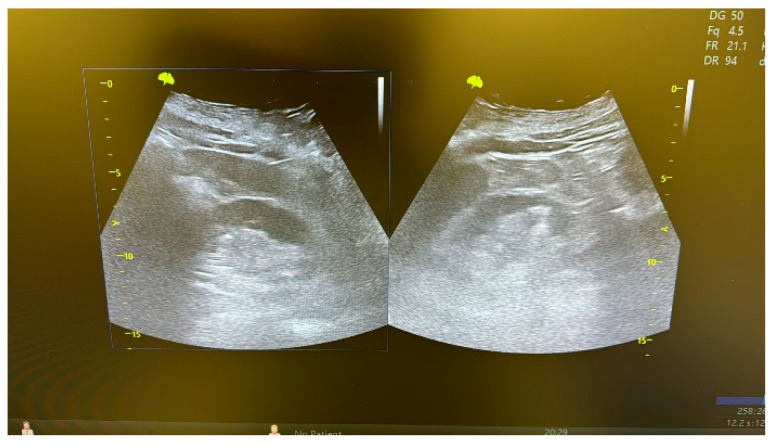
Ultrasound evaluation of the kidneys during follow-up.

The double-J stent was removed 6 months postoperatively, following an endoscopic evaluation of the reconstructed ureter (Figure 10). At the 6-month follow-up, a CT scan or abdominal ultrasound (Figure 11) was performed to confirm the absence of stricture recurrence and normal ipsilateral renal function.

During a mean clinical follow-up of 38 months, all patients remained symptom-free. However, one patient experienced ureteral stricture recurrence following LUU for a lumbar stricture, and required permanent placement of a double-J stent. The overall success rate of the procedure was 88.88%.

Table 1 summarizes the demographic and preoperative clinical characteristics of the patients. The minimally invasive approach was technically feasible in all patients, without needing conversion. The mean length of the ureteral stricture was 19 mm (range: 5–40 mm), calculated using the CT scan. Intraoperative and postoperative data are presented in Table 2. The median operating room time was 105 min, and the median hospital stay was 4 days. The median catheterization time and hospital stay were similar in all patients, and no significant preoperative complications occurred. The double-J stent was removed at 6 months after surgery, after an endoscopic evaluation of the procedure (Figure 10). A CT scan or an abdominal ultrasound (Figure 11) was performed 6 months later to confirm the absence of stricture recurrence and normal ipsilateral renal function. All patients remained symptom-free during the 38-month clinical follow-up.

We report a case of ureteral stricture recurrence following LUU for lumbar stricture, with the patient remaining with a permanent double-J. Our success rate was 88.88%.

## 4. Discussion

Iatrogenic ureteral stenosis is a relatively common complication that can occur following urological procedures, including open, laparoscopic, robotic, or endoscopic surgery [8]. Fiori’s letter underscores the negative aspects of ureteroscopy, stressing that previous surgeries can lead to ureteral stenosis and other complications that jeopardize renal function [9]. Fibrosis and scarring at the prior surgical site make ureteral redo surgery particularly challenging.

Laparoscopic and robot-assisted techniques have been developed as alternatives to open surgery, aiming to minimize morbidity and shorten hospital stays. Advances in surgical technology have enabled the integration of minimally invasive procedures into routine clinical practice, including complex reconstructive surgeries that were previously performed exclusively through open surgery.

The first successful LUU was conducted in 1992 by Nezhat et al. [3]. In a retrospective trial comparing open and laparoscopic ureteral reconstruction, Simmons et al. found that the laparoscopic approach resulted in reduced blood loss and shorter hospital stays, while maintaining comparable patency and complication rates [10]. Similarly, De Cicco et al. reported equivalent recurrence rates between laparoscopic and open approaches, with success rates exceeding 90% [11]. However, strictures may recur up to one year postoperatively, highlighting the need for long-term follow-up. While recent reports on laparoscopic ureteral reconstruction demonstrate promising outcomes, multicenter, randomized studies with extended follow-up periods are required to validate these findings [12].

Laparoscopic ureteral reconstruction remains a technically demanding procedure, requiring advanced surgical skills and a long learning curve.

Although laparoscopy mimics open surgery, it lacks flexibility, making certain steps more challenging. The advent of robotic platforms has significantly improved ureteral reconstructive surgery by enhancing surgical precision, dexterity, and visualization. The Da Vinci robotic system has mitigated many of the technical difficulties of laparoscopy, particularly in the reconstruction phase. Robotic surgery offers advantages such as improved ureteral mobilization, enhanced intracorporeal suturing, and facilitated antegrade double-J stent placement. It also provides superior access to the upper urinary tract, especially in cases involving prior ureteral resection, without the limitations of laparoscopic instruments or the larger incisions required for open surgery [13]. Minimally invasive surgery aims to reproduce all the key principles of open surgery, while ensuring a watertight but not ischemic, tension-free, and well-vascularized anastomosis to avoid further recurrences.

Our experience confirms that both robotic and laparoscopic approaches are feasible and well tolerated for ureteral reconstruction, yielding excellent perioperative and functional outcomes.

Hemal et al. retrospectively analyzed seven patients undergoing RAUU and reported a mean operative time of 110 min, an estimated blood loss of 50 mL, and a hospital stay of 3 days. No recurrences were observed at a mean follow-up of 28 months [14]. Buffi et al. [5] reported on 17 patients undergoing RAUU, noting a mean operative time of 150 min and a 94% success rate, with no complications exceeding Clavien–Dindo Grade II. A comparative study of 126 patients demonstrated that robotic ureteral reconstruction was associated with shorter operative and hospitalization times and lower inflammatory response markers than laparoscopic reconstruction [15].

A retrospective study by Schiavina et al. found that while estimated blood loss was lower in the robotic group, other surgical outcomes were similar when comparing robotic and laparoscopic approaches for iatrogenic ureteral stenosis. The study concluded that minimally invasive ureteral reconstruction is safe and feasible, with a high success rate (95%) at a median follow-up of 27 months [16]. Tracey et al. [17] reported similar findings when comparing robotic and open ureteral reconstruction, noting reduced blood loss and shorter hospital stays with robotic surgery.

A large single-center retrospective study of 55 patients undergoing robotic ureteral reconstruction observed three failures (5.3%) and two postoperative complications (3.6%) at a mean follow-up of 181 days. Several reconstruction methods were used, such as ureteroureterostomy, ureteral reimplantation with psoas hitch, and the Boari flap. The average surgery took 221 min, and 50 mL of blood was lost. The median hospital stay was 1.6 days [18].

The decision to use a refluxing versus a non-refluxing anastomotic technique remains at the discretion of the surgeon, as studies have shown similar rates of stenosis and renal function preservation, regardless of technique [19,20]. Marien et al. analyzed 31 cases of robotic extravesical ureteroneocystostomy, reporting complete clinical and radiological improvement [21]. Another study involving 45 patients who underwent robotic ureteral reimplantation with or without a psoas hitch demonstrated a 94% success rate, with no conversions to open surgery and a major complication rate of 3.6% (Clavien grade > 3) [18].

The increasing adoption of robotic-assisted ureteral reconstruction has been associated with improved surgical precision, enhanced visualization, and greater dexterity, leading to favorable outcomes in high-volume centers. Hospitals performing a higher volume of robotic procedures tend to achieve better results, reinforcing the importance of surgical expertise in optimizing patient outcomes.

Despite these advancements, our study has some limitations. The relatively small number of robotic cases limits direct comparisons between laparoscopic and robotic approaches. We used the robotic technique for a post-pancreaticoduodenectomy ureteral stricture, anticipating extensive fibrosis and requiring improved dexterity and precision. However, without a larger cohort of robotic cases, it is difficult to generalize findings. Additionally, the median follow-up duration of 38 months is relatively short and variable.

Another limitation is the absence of a direct comparison with open ureteral reconstruction. While minimally invasive approaches have demonstrated reduced blood loss, shorter hospital stays, and faster recovery times, the technical demands and learning curve remain significant. Prior studies, such as that by Simmons et al. [10], have reported similar patency and complication rates between laparoscopic and open approaches, raising concerns about the cost-effectiveness and accessibility of robotic-assisted surgery.

Our overall success rate was 88.88%, with only one case of stricture recurrence requiring permanent double-J stenting, underscoring the challenges of redoing ureteral reconstruction. Future studies with larger patient cohorts, extended follow-up periods, and randomized comparisons between surgical techniques are essential to establish the optimal approach for managing iatrogenic ureteral strictures.

## 5. Conclusions

Robotic and laparoscopic reconstructive ureteral surgery performed by expert surgeons in a tertiary referral center is a safe and feasible procedure, with a low overall rate of postoperative complications. This minimally invasive approach may be a final alternative to endoscopic procedures for permanent stenting, autotransplantation, or distal ureteral reconstruction with a bladder flap in the Cassati Boari fashion. While robotic assistance offers surgical precision and suturing advantages, more research is needed to determine its long-term benefits over laparoscopy. More multicenter studies and randomized controlled trials are required to improve surgical algorithms, find the best patients, and prove that minimally invasive techniques are better for complex ureteral reconstruction.

## Figures and Tables

**Table 1:** Demographic characteristics.

Patients’ Characteristics	No
Patients, no. (male/female)	2/7
Mean patient age, yr (range)	42 (23–64)
Patients with right ureter stricture, no.	4
Patients with left ureter stricture, no.	5
Concomitant stones, no.	1
Median body mass index (range)	26.4 (21.7–29.1)
Symptoms	9
Mean ureteral stricture length, mm	19 (5–40)

**Table 2:** Postoperative outcomes.

Outcomes	
Operative time, min, median (IQR)	105 (20.0)
Postoperative complications, no. (type)	0
Bladder catheterization time, days, median—lumbar and iliac stricture	12 (10–14)
Bladder catheterization time, days, (median) ureteral reimplantation	21 (19–24)
Hospital stay, days, median (range)	4 (3–5)
Recurrence, no. (%)	1 (11.11)
Follow-up, mo, median (IQR)	38 (36.0)

Interquartile range (IQR).

## Data Availability

Anonymized data may be available from the corresponding author upon reasonable request.
